# The effective threshold dose of etanercept in patients with methotrexate-resistant rheumatoid arthritis

**DOI:** 10.1007/s10067-023-06659-9

**Published:** 2023-07-07

**Authors:** Fangfang Chen, Yitian Lang, Shikai Geng, Xiaodong Wang, Liangjing Lu, Shuang Ye, Le Zhang, Ting Li

**Affiliations:** 1 Department of Rheumatology, Ren Ji Hospital, Shanghai Jiaotong University School of Medicine, Shanghai, People’s Republic of China; 2 Department of Pharmacy, Ren Ji Hospital, Shanghai Jiaotong University School of Medicine, Shanghai, China; 3 Department of Pharmacy, Huangpu Branch, Shanghai Ninth People’s Hospital, Shanghai Jiaotong University School of Medicine, Shanghai, China

**Keywords:** Cost-effectiveness, Effective cumulative dose, Etanercept, Rheumatoid arthritis, Saturated dose

## Abstract

**Introduction:**

The therapy of rheumatoid arthritis (RA) was advanced by biological agents, yet costly. This study aims to identify the effective threshold dose of etanercept (ENT) and cost-effectiveness in methotrexate (MTX)-resistant RA in real world.

**Methods:**

Eligible patients had an inadequate response (DAS28-ESR > 3.2) to initial MTX monotherapy, and subsequently received etanercept. The effective cut-off value of cumulative dose was identified to maintain remission response (DAS28-ESR < 2.6) at month 24 by using restricted cubic splines. Remission rate, low disease activity (LDA) rate, glucocorticoid exposure, safety, and cost-effectiveness were compared between the saturated and non-saturated dose groups divided by the cut-off dose.

**Results:**

Seventy-eight (14.2%) of 549 enrolled patients were eligible, and 72 patients completed follow-up. The 2-year cumulative cut-off dose that maintained remission response at 24 months was 1975 mg. And the recommended threshold dosing strategy of etanercept was twice weekly (BIW) for the first 6 months, every week (QW) for the next 6 months, and every 2 weeks (Q2W) and every month (QM) for the second year. Greater net changes in DAS28-ESR score were observed in the ENT saturated dose group than in the non-saturated dose group (average change 0.569, 95%CI 0.236–0.901, *p* = 0.001). The proportion of patients achieving remission (27.8% vs 72.2%, *p* < 0.001) and LDA (58.3% vs 83.3%, *p* = 0.020) in the non-saturated group was both significantly lower than that in the saturated group at 24 months. The incremental cost-effectiveness ratio of the saturated group referred to the non-saturated group was 5791.2 $/QALY.

**Conclusions:**

In refractory RA patients, the effective cumulative cut-off dose of etanercept for sustained remission at 24 months was calculated as 1975 mg, and receiving saturated dose was more effective and cost-effective than with non-saturated dose.

**Table:** 

**Key Points** *• The effective cumulative cut-off dose of etanercept for sustained remission at 24 months in RA patients is calculated as 1975 mg.* *• Receiving saturated dose of etanercept is more effective and cost-effective than with non-saturated dose in refractory RA patients.*

**Supplementary Information:**

The online version contains supplementary material available at 10.1007/s10067-023-06659-9.

## Introduction

Rheumatoid arthritis (RA), a systemic inflammatory disease, could lead to significant joint destruction and functional disability due to improper treatment [1]. It is critical to control disease activity to limit structural damage and functional impairment. In recent years, biological treatment has greatly advanced and revolutionized the therapy of RA [5]. However, tapering strategy of biological agents has emerged as an important consideration after achieving remission, in light of the adverse event and economic burden [6]. It brings to another challenging problem: once the treatment goal is reached, that is, remission or at least low disease activity (LDA), how should the therapy taper or even stop to avoid patient overtreatment.

Many studies have begun to explore that when patients reach LDA or remission, they can consider reducing or even stopping biological disease-modifying antirheumatic drugs (bDMARDs) [9]. Based on the evidence from these studies, the European League Against Rheumatism (EULAR) and the American College of Rheumatology (ACR) include the choice of dose reduction in their latest guidelines, the core content of which is that “maintaining the treatment target does not necessarily mean maintaining the treatment intensity” [12]. Therefore, the optimal reduction strategy of biological agents has become an important aspect for RA patients to achieve remission.

Some randomized controlled and observational studies for the withdrawal of tumor necrosis factor inhibitors (TNFi) in rheumatoid arthritis have been conducted [15], suggesting that tapering is feasible. However, no cohort study has yet been reported to assess dose reduction or cumulative dose thresholds, and consequently, evidence on tapering strategies in the real world for RA remains lacking. Despite tapering strategies that have been proposed based on previous studies, patients in actual clinical practice often encounter poor treatment adherence and financial constraints which prevent them from following the established tapering schemes strictly. Therefore, it may be more pragmatic to identify a balance point during long-term follow-up, wherein maintaining a certain level of dose accumulation over a specific period of time could potentially result in favorable treatment outcomes. In order to implement tapering optimization, we conducted an observational study from a real-world cohort to identify the effective cut-off cumulative dose of etanercept in patients with methotrexate (MTX)-resistant RA, and give the points for correlation between tapering strategies and treat-to-target (T2T) principle [20].

## Materials and methods

### Study design

Eligible patients’ data were extracted when the following inclusion criteria were met: (1) all patients fulfilled the 2010 criteria for RA of the American College of Rheumatology or European League Against Rheumatism [21]; (2) they had an inadequate response to initial MTX monotherapy, defined as DAS28-ESR > 3.2 (28-joint disease activity score using erythrocyte sedimentation rate) after receiving MTX for at least 6 months with a stable route of administration and more than 10 mg weekly for at least 3 months prior to the baseline visit [22]; (3) and subsequently using etanercept 25 mg twice weekly (BIW) by subcutaneous injection; (4) had at least 24 months of follow-up after giving etanercept. Patients were excluded if they were illiterate, had severe mental disorders, had serious physical constraints, or were over the age of 85. The tapering and increase of etanercept or switch to another biologic agent were allowed according to the T2T recommendations. Conventional synthetic disease-modifying antirheumatic drugs (csDMARDs), non-steroidal anti-inflammatory drugs (NSAIDs), and glucocorticoids (GCs) were allowed to administrate at stable doses if needed. Tapering of GCs or csDMARDs was allowed according to routine practice, but not to be changed within 2 weeks before assessment.

### ROC analysis

The cumulative dose for 24 months of each individual was recorded, and the effective cut-off of dose was calculated to identify the remission response (DAS28-ESR < 2.6) at 12 months [23]. The appropriate cut-off value of 12-month cumulative dose was estimated by constructing a receiver operating characteristics (ROC) curve. The Youden index is a measure of the accuracy of the model, which is used to determine the optimal cut-off value. Correlations were calculated by using ROC analyses and the point estimates of the area under the curve (AUC) and surrounding confidence interval (CI) to verify that the lower limit of CI was above 0.5. The sensitivity (true positive rate) represents the proportion of observations that were predicted to be positive when they were positive. In contrast, the 1-specificity (false positive rate) represents the proportion of observations that were predicted to be positive while they were negative.

### Outcome assessment

The outcome assessment included the proportion of patients having reached at least one remission/LDA over 2 years, average daily exposure to glucocorticoid, the proportion of patients’ withdrawal from GCs, and cost-effectiveness by the available extended 2-year data in patients. DAS28 scores, laboratory values, quality of life assessments by EuroQol five-dimensions questionnaire (EQ5D), and medications during the follow-up were recorded [24]. The changes of DAS28 over 24-month follow-up were also analyzed. Drug safety was assessed by self-reported records among the overall population at each follow-up record. The occurrences of disease flare were defined as follows: (1) DAS28 > 3.2; (2) increase dose in DMARDs and/or GCs; (3) switch to another biologic agent.

### Cost-effectiveness analysis

We developed a decision tree model to assess the mean between-group difference in costs and quality-adjusted life years (QALYs) gained over 24 months among the patients who completed a 2-year follow-up without switching therapy regimens. Based on the Chinese time-trade-off method, the EQ-5D questionnaire responses yielded health utility values, from which we calculated cumulative QALYs [24]. An incremental cost-effectiveness ratio (ICER) was calculated by dividing the cost difference by the QALY difference in per pair of treatment schemes [26]. A 10,000 iteration Monte Carlo simulation was implemented to get the cost-effectiveness acceptability curve (CEAC) to analyze the preferred treatment group in a range of willingness-to-pay (WTP) threshold intervals. And the cost-effectiveness plane was also developed to view the distribution between each iteration and WTP threshold. The WTP thresholds under the per capita gross domestic product (GDP, around $12,552.08 in 2021) of China was considered cost-effective. All costs sourced from China in this study were converted into US dollars ($1 = RMB 6.4512, average exchange rate for 2021).

### Ethics

This observational cohort was established following the OPCSP trial (ClinicalTrials.gov ID: NCT03024307), a randomized trial on a compliance improvement program among rheumatic patients by a multidisciplinary team. The research protocol was approved by Shanghai Jiaotong University School of Medicine, Renji Hospital Ethics Committee (approval No. [2016] 216 K). All participating patients provided written informed consent.

### Statistical analysis

Categorical variables were described by frequency and percentage, while continuous variables were described by mean ± standard deviation or median and quartile. The comparisons between the study groups were performed by Student’s *t*-test for continuous variables and the chi-square test (or Fisher’s exact test) for categorical variables. We also used restricted cubic splines (RCS) with four knots to flexibly model the association of predicted cumulative dose with sustained remission response [27]. RCS can identify non-linear relationships, provide more precise effect size estimates, facilitate thresholds identification, and improve predictive accuracy. The change of DAS28 over time was compared by specifying a linear mixed model with treatment and time as fixed factors and was performed with baseline DAS28 score as a covariate. As the visit schedules were slightly different between both groups, only baseline, 6-month, 12-month, and 24-month data were included for the analysis of the secondary outcomes. All statistical calculations were performed using the statistical software package IBM SPSS version 26.0 (IBM, Armonk, NY, USA). For all tests, *p* < 0.05 was considered to be significant.

## Results

### Cohort baseline

Seventy-eight out of 549 patients of the cohort were eligible participants, and 72 patients completed 2-year follow-up. Baseline demographics and clinical characteristics of patients were summarized in Table 1. The average disease duration was 8.6 years and all of them had a diagnosis of RA longer than 1 year. The proportion of patients with positive ACPA and/or RF was over 80%. The average disease activity was 5.0 according to DAS28-ESR and 20 patients (27.7%) were in high disease activity. In terms of concomitant medication, the average dose of MTX was 10 mg weekly. Twenty-eight patients (38.8%) received GCs in this study at an average daily dose of 8.5 mg.

**Table 1:** Demographic and clinical characteristics of patients at baseline

Characteristics	Overall (*n* = 72)
Age, mean (S.D.), yrs	56.8 (13.8)
Female, *n* (%)	56 (77.7)
BMI, mean (S.D.)	22.4 (2.6)
Smoking history, *n* (%)	6 (8.3)
Drinking history, *n* (%)	4 (5.5)
Disease duration, mean (S.D.), yrs	8.6 (6.8)
Comorbidities, *n* (%)	33 (45.8)
0	39 (54.1)
1	17 (23.6)
2	10 (13.8)
≥ 3	6 (8.2)
ACPA + *, *n* (%)	48 (80.0)
RF + **, *n* (%)	52 (88.1)
VAS pain, mean (S.D.)	6.4 (2.1)
DAS28-ESR, mean (S.D.)	5.0 (1.0)
DAS28-CRP, mean (S.D.)	4.4 (0.9)
HDA rate, *n* (%)	20 (27.7)
EQ-5D index, mean (S.D.)	0.7 (0.1)
GCs usage, *n* (%)	28 (38.8)
GCs dosage#, mean (S.D.), mg	8.5 (6.1)
Weekly dose of MTX, mean (S.D.), mg	11.1 (2.0)
Prior DMARDs, mean (S.D.)	2.3 (1.2)
bDMARDs, mean (S.D.)	0.2 (0.5)
Use of NSAIDs, *n* (%)	12 (16.6)

*BMI*, body mass index; *MTX*, methotrexate; *ACPA*, anticitrullinated protein antibody; *RF*, rheumatoid factor; *VAS pain*, visual analogue scale for pain (0–10); *DAS28-ESR*, disease activity score in 28 joints using erythrocyte sedimentation rate; *DAS28-CRP*, disease activity score in 28 joints using c-reactive protein; *HDA*, high disease activity; *EQ-5D*, EuroQol five-dimensions questionnaire; *GCs*, glucocorticoids; *DMARDs*, disease-modifying antirheumatic drugs; *bDMARDs*, biological DMARDs; *NSAIDs*, non-steroidal anti-inflammatory drugs

^*12 missing data was excluded in calculating the positive proportion^

^**13 missing data was excluded in calculating the positive proportion^

^#Average daily dose of GCs was calculated in prescribed patients^

### Cut-off value of cumulative dose

**Fig. 1:**
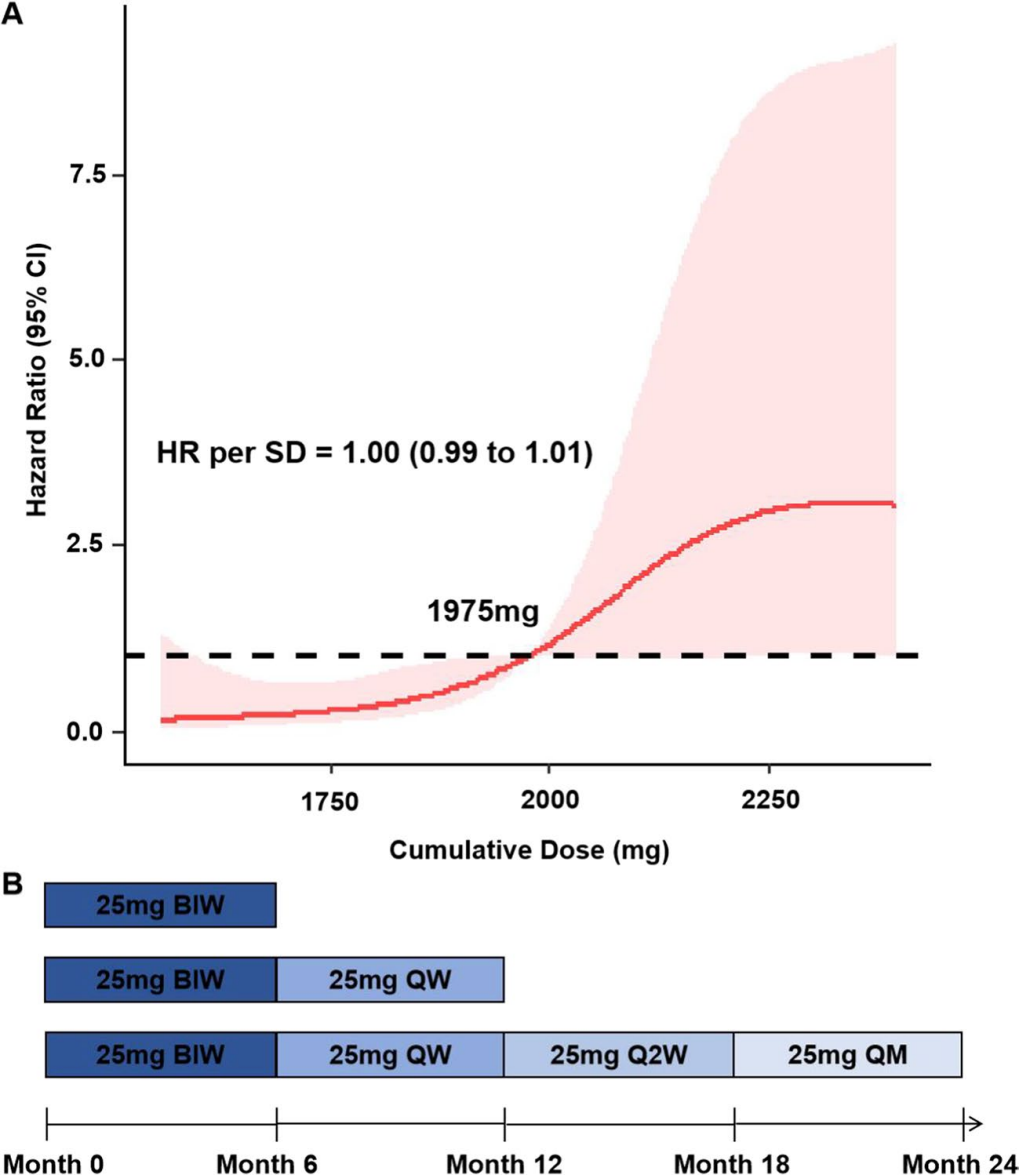
Dose–response association between cumulative dose and sustained remission response at 24 months using restricted cubic splines and recommended frequency of use. ROC, receiver operating characteristics; AUC, area under the curve; BIW, twice weekly; QW, every week; Q2W, every 2 weeks; QM, every month

We used restricted cubic splines to flexibly model and visualize the dose–response relation of the predicted cumulative dose with sustained remission response without flare (as defined in the “Outcome assessment”) at 24 months in RA patients (Fig. 1A). The probability of the sustained remission response was relatively flat until around 1975 mg of the predicted cumulative dose and then started to increase rapidly afterward (*p* for non-linearity = 0.009). Above 1975 mg, the hazard ratio per standard deviation higher predicted cumulative dose was 1.00 (0.99 to 1.01). Seventy-two participants who completed 2-year follow-up were divided into saturated dose group (*n* = 36) and non-saturated dose group (*n* = 36) by the cut-off dose (Supplementary Figure S1).

The 6-month and 1-year cumulative cut-off doses of 1075 mg and 1625 mg for remission response were identified from the ROC curve; area under ROC was 0.613 (95%CI 0.472–0.754, *p* = 0.072) and 0.730 (95%CI 0.069–0.851, *p* = 0.001) (Supplementary Figure S2). Youden’s index was calculated as *J* = 0.195 and *J* = 0.369 with the sensitivity of 45.5% and 56.0%, and specificity of 74.0% and 80.9% respectively (Supplementary Table S1).

According to the cumulative cut-off dose, the recommended threshold dosing frequency of etanercept for the first 6 months, the next 6 months, and the second year were twice weekly (BIW), every week (QW), every 2 weeks (Q2W), and every month (QM) respectively (Fig. 1B).

### Efficacy

**Fig. 2:**
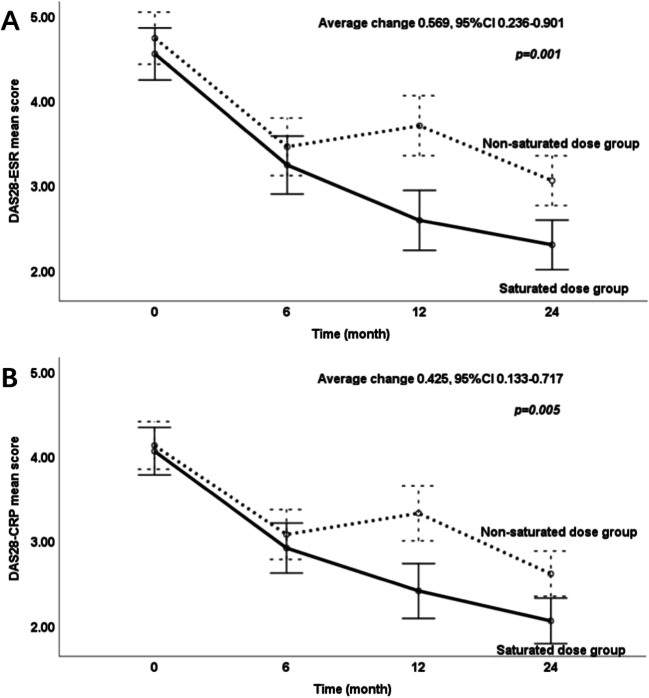
Changes of DAS28 score over 2 years. The solid line represents the saturated dose group, and the dashed line represents the non-saturated dose group. CI, confidence interval; DAS28-ESR, disease activity score in 28 joints using erythrocyte sedimentation rate; DAS28-CRP, disease activity score in 28 joints using c-reactive protein

A significant difference of the net changes of DAS28-ESR score over 2 years was observed, a greater average change in DAS28-ESR reduction in the saturated dose group than in the non-saturated dose group (average change 0.569, 95%CI 0.236–0.901, *p* = 0.001) (Fig. 2A). Greater net changes of DAS28-CRP score were also observed in the saturated dose group than in the non-saturated dose group (average change 0.425, 95%CI 0.133–0.717, *p* = 0.005) (Fig. 2B).

The proportion of patients achieving remission (27.8% vs 72.2%, *p* < 0.001) and LDA (58.3% vs 83.3%, *p* = 0.020) in the non-saturated group was both significantly lower than that in the saturated group at 24 months. In comparison, 16.5% of the non-saturated group achieved sustained LDA and 72.2% of those assigned to the saturated group had achieved sustained LDA at 24 months (*p* < 0.001). And the sustained remission rates in the non-saturated group were both significantly lower than those in the saturated group at 24 months (8.3% vs 52.8%, *p* < 0.001).

The remission (16.7% vs 52.8%, *p* = 0.001) and LDA rate (25.0% vs 75.0%, *p* < 0.001) in the non-saturated group were both significantly lower those than in the saturated group at 12 months. However, there were no significant differences between the two groups in remission and LDA rate at 6 months. At the endpoint of 24 months, among the 72 patients available for analysis, daily dose of GCs (4.0 vs 5.8 mg, *p* = 0.316) was not significantly (Table 2).

**Table 2:** Treatment efficacy during follow-up

Measure	Month 0			Month 6			Month 12			Month 24		
	Saturated (*n* = 36)	Non-saturated (*n* = 36)	*p* value	Saturated (*n* = 36)	Non-saturated (*n* = 36)	*p* value	Saturated (*n* = 36)	Non-saturated (*n* = 36)	*p* value	Saturated (*n* = 36)	Non-saturated (*n* = 36)	*p* value
DAS28-ESR, mean (S.D.)	4.6 (0.9)	4.7 (1.0)	0.396	3.2 (1.0)	3.5 (1.0)	0.376	2.6 (1.1)	3.7 (1.1)	< 0.001	2.3 (0.9)	3.1 (0.9)	0.001
DAS28-CRP, mean (S.D.)	4.1 (0.8)	4.1 (0.9)	0.735	3.0 (0.9)	3.1 (0.9)	0.446	2.4 (0.8)	3.3 (1.1)	< 0.001	2.1 (0.7)	2.6 (0.9)	0.005
VAS pain, mean (S.D.)	5.9 (2.2)	6.1 (1.9)	0.731	3.3 (1.9)	4.3 (2.0)	0.029	2.7 (2.1)	4.0 (2.2)	0.013	2.0 (1.5)	3.1 (2.0)	0.010
Remission rate, *n* (%)	0	0	/	14 (38.9)	8 (22.2)	0.125	19 (52.8)	6 (16.7)	0.001	26 (72.2)	10 (27.8)	< 0.001
Sustained remission rate, *n* (%)	/	/	/	/	/	/	/	/	/	19 (52.8)	3 (8.3)	< 0.001
LDA rate, *n* (%)	0	0	/	18 (50.0)	13 (36.1)	0.234	27 (75.0)	9 (25.0)	< 0.001	30 (83.3)	21 (58.3)	0.020
Sustained LDA rate, *n* (%)	/	/	/	/	/	/	/	/	/	26 (72.2)	7 (16.5)	< 0.001
EQ5D, mean (S.D.)	0.727 (0.1)	0.698 (0.1)	0.377	0.750 (0.2)	0.699 (0.2)	0.174	0.799 (0.1)	0.698 (0.2)	0.016	0.812 (0.1)	0.693 (0.2)	0.011
GCs usage, *n* (%)	10 (27.8)	18 (50.0)	0.053	8 (22.2)	19 (52.8)	0.007	9 (25.0)	20 (55.6)	0.008	6 (16.7)	17 (47.2)	0.005
GCs tapering and withdrawal, *n* (%)	/	/	/	4 (40.0)	5 (27.8)	0.809	5 (41.7)	5 (23.8)	0.496	7 (70.0)	6 (33.3)	0.142
GCs dose*, mean (S.D.), mg	6.8 (3.3)	9.5 (7.1)	0.258	4.1 (3.2)	7.3 (4.7)	0.058	4.3 (4.2)	7.0 (4.7)	0.104	4.0 (6.1)	5.8 (4.2)	0.316
Use of NSAIDs, *n* (%)	6 (16.7)	6 (16.7)	1.000	6 (16.7)	0	0.025	3 (8.3)	0	0.239	2 (5.6)	0	0.493

*Notes*: *Average daily dose of GCs was calculated in prescribed patients

*DAS28-ESR*, disease activity score in 28 joints using erythrocyte sedimentation rate; *DAS28-CRP*, disease activity score in 28 joints using c-reactive protein; *VAS pain*, visual analogue scale for pain (0–10); *LDA*, low disease activity; *EQ-5D*, EuroQol five-dimensions questionnaire; *GCs*, glucocorticoids; *NSAIDs*, non-steroidal anti-inflammatory drugs

### Safety analysis

There were no serious adverse effects observed during the follow-up, and no difference in the proportion of patients experienced comorbidities (50.0% vs 63.9%, *p* = 0.234) or adverse effects (19.4% vs 27.8%, *p* = 0.405) across treatment groups (Table 3). The most common comorbidity in our study was cardiovascular diseases (30.6%), followed by digestive system diseases (23.6%), hematological system diseases (18.1%), osteoporosis (16.7%), and renal disease (16.7%). The two most common recorded adverse effects were infections (8.3%) and hepatotoxicity (8.3%), followed by gastrointestinal response (5.6%) and leukopenia (1.4%).

**Table 3:** Safety outcomes over 24 months (*n* = 72)

Measure	Saturated (*n* = 36)	Non-saturated (*n* = 36)	*p* value
Comorbidities, *n* (%)	18 (50.0)	23 (63.9)	0.234
0	18 (50.0)	13 (36.1)	
1	5 (13.8)	9 (25.0)	
2	6 (16.6)	5 (13.8)	
≥ 3	7 (19.4)	9 (25.0)	
Cardiovascular diseases	12 (33.3)	10 (27.8)	0.609
Digestive system diseases	8 (22.2)	9 (25.0)	0.781
Hematological system diseases	9 (25.0)	4 (11.1)	0.126
Osteoporosis	5 (13.9)	7 (19.4)	0.527
Renal disease	3 (8.3)	9 (25.0)	0.058
Musculoskeletal diseases	4 (11.1)	7 (19.4)	0.326
Endocrine system	3 (8.3)	4 (11.1)	1.000
Respiratory diseases	0	2 (5.6)	0.493
Nervous system	1 (2.8)	1 (2.8)	1.000
Adverse effects, *n* (%)	7 (19.4)	10 (27.8)	0.405
Infections	3 (8.3)	3 (8.3)	1.000
Hepatotoxicity	2 (5.6)	4 (11.1)	0.674
Gastrointestinal response	1 (2.8)	3 (8.3)	0.614
Leukopenia	1 (2.8)	0	1.000

### Cost-effectiveness

Medication costs and QALYs for each treatment group are presented in Table 4. The medication costs for 2 years in the ENT non-saturated dose group was $3320.66, and $4450.51 in the saturated dose group. The patients gained an average QALYs of 1.391 in the non-saturated group and 1.587 in the saturated group. The ICER yielded by the saturated dosing therapy was $5791.2 per QALY compared with the non-saturated dosing therapy, within the WTP threshold set as the per capita GDP of China.

**Table 4:** Base-case analysis over 24 months

Measure	Non-saturated dose group (*n* = 36)	Saturated dose group (*n* = 36)
Medication costs (US$)	3320.66	4450.51
Δ Costs (US$)	Reference	1129.85
QALYs	1.391	1.587
Δ QALYs	Reference	0.195
ICER ($/QALY)	Reference	5791.2

*ICER*, incremental cost-effectiveness ratio; *QALYs*, quality-adjusted life years; *Average C/E*, average cost-effectiveness ratio

**Fig. 3:**
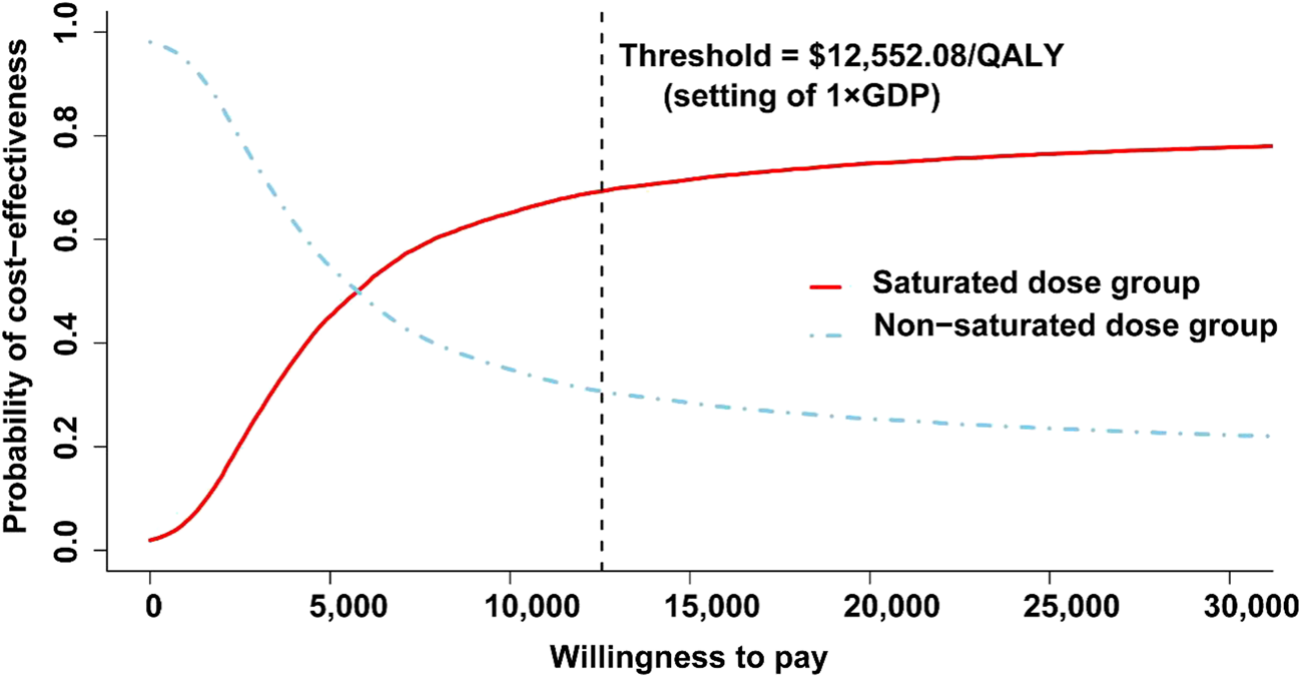
Cost-effectiveness acceptable curve of two groups. QALY, quality-adjusted life year, GDP, gross domestic product

A 10,000 iteration Monte Carlo simulation was conducted to evaluate the robustness of cost-effectiveness analysis. The output was displayed in the form of cost-effectiveness acceptable curve (CEAC) and cost-effectiveness plane (CE plane). The CEAC (Fig. 3) showed the saturated group was 69.3% of being cost-effective at the $12,552.08/QALY threshold. And the non-saturated group was 30.7% at the same threshold. The CE plane also revealed the results (Supplementary Figure S3). Each dot represents one output. Around 6930 outputs were below the WTP threshold in the saturated dose group. In the setting of 1 × GDP, the results of the saturated group showed high probability of being cost-effective regimen than the non-saturated dose group.

## Discussion

According to the results of restricted cubic splines, we obtained the critical value of 2-year cumulative dose that can maintain the compliance of remission at 24 months was 1975 mg. Further combining with the cumulative cut-off dose at 6 and 12 months from the ROC curve, we obtained the recommended threshold dosing frequency of etanercept. It suggested that at least a certain cumulative dose of biological agents is necessary for the maintenance of long-term therapeutic effect in early treatment. And it is more conducive to the long-term control of the disease and improves the quality of life of patients.

Achievement of sustained remission or LDA is the overarching treatment goal in patients with RA to reduce the risk of joint damage and disability [20]. Based on our results, the ENT saturated dosing therapy was significantly better than the non-saturated dosing therapy in respect of remission/LDA response, more importantly, sustained remission/LDA rate. Compared with the results of our previous study [28], the proportion of patients achieving sustained remission and LDA at 24 months in the non-saturated group was 8.3% and 16.5% respectively, while 25.3% and 53.2% in the MTX + HCQ group. It partly suggested that the combination of csDMARDs was more benefit to the long-term prognosis of patients if the adequate dose or course of bioDMARDs treatment could not be guaranteed.

We also found that 52.8% of the patients benefited from the tapering regimen; that is, remission response could be maintained at 24 months, suggesting that a significant proportion still need adequate TNF therapy. In the STRASS study (Spacing of TNF-blocker injections in Rheumatoid ArthritiS Study), patients were randomized to either continuing full-dose TNF inhibitor (etanercept or adalimumab) or tapering it by spacing the injection interval. Thirty-nine percent of the patients stopped the TNF inhibitor in the tapering arm while maintaining the remission status [18]. Smolen et al. investigated the effect of stopping etanercept; 43% of the patients remained in low disease activity over 1 year [19]. Meanwhile, the proportion of patients achieving remission and LDA at 6 months among the saturated group was 38.9% and 50.0% respectively. From the result of previous report, the remission and LDA rate of combination of csDMARDs were 12.7% and 24.8% [29]. Therefore, biological agents still showed advantages in early response to refractory RA patients. However, we still need further research to compare the efficacy of MTX plus HCQ or etanercept.

To our knowledge, rare study have been reported on the cost-effectiveness of a dose optimization strategy of TNFi therapy in RA patients. We found that the ICER yielded by the saturated dosing therapy was $5791.2 per QALY compared with the non-saturated dosing therapy, within the WTP threshold set as the per capita GDP of China. The medication costs were significantly reduced compared with the result of optimization and standardized control treatment ($17,085 vs $29,699) in the DRESS study, also reported that disease activity-guided dose optimization would be a more cost-effective approach than standardized control treatment.

In this study, we observed more patients received multidisciplinary care in the saturated dose group than in the non-saturated dose group. Although there are many options for the treatment of RA, due to its course migration, difficulty in remission, the patient’s compliance is poor, which leads to the risk of recurrence and damage to disease control. Effective and comprehensive disease management is the key to ensuring the treatment effect and achieving long-term remission. Thus, optimal disease management could enhance patient compliance, which is critical to controlling disease activity.

Our study has several limitations. First, the sample size of this study was small. It is difficult to find patients who use a single biological agent to finish the 2-year follow-up in the real world. Moreover, the assessment of the long-term impact of biological agents’ reduction is not comprehensive, such as the lack of adequate imaging evaluation evidence. Several studies have investigated the long-term feasibility and risks of TNF-blocker reduction or discontinuation in established RA, especially on radiographic progression [10]. In this study, the current results are still prominent and meaningful and give instructive advice for trying to answer the specific maintenance scheme of biological agents for long-term treatment of RA.

## Conclusions

In patients with refractory rheumatoid arthritis, the effective cumulative cut-off dose of etanercept for sustained remission response at 24 months was identified as 1975 mg. Receiving saturated dose etanercept was superior in efficacy and cost-effectiveness to non-saturated dose.

### Supplementary Information

Below is the link to the electronic supplementary material.

Supplementary file1 (PDF 368 KB)

Supplementary file2 (DOCX 35 KB)

## Data Availability

The datasets used or analyzed during the current study are available from the corresponding author on reasonable request.
