# Correction to: The effective threshold dose of etanercept in patients with methotrexate-resistant rheumatoid arthritis

**DOI:** 10.1007/s10067-023-06727-0

**Published:** 2023-08-09

**Authors:** Fangfang Chen, Yitian Lang, Shikai Geng, Xiaodong Wang, Liangjing Lu, Shuang Ye, Le Zhang, Ting Li

**Affiliations:** 1 Department of Rheumatology, Ren Ji Hospital, Shanghai Jiaotong University School of Medicine, Shanghai, People’s Republic of China; 2 Department of Pharmacy, Ren Ji Hospital, Shanghai Jiaotong University School of Medicine, Shanghai, China; 3 Department of Pharmacy, Huangpu Branch, Shanghai Ninth People’s Hospital, Shanghai Jiaotong University School of Medicine, Shanghai, China

**Correction to: Clinical rheumatology**

https://doi.org/10.1007/s10067-023-06659-9

The affiliations in the published version of the attached above article was incorrectly designated to the authors. The correct presentation are as follows:

**Fangfang Chen** ^**1**^ **, Yitian Lang** ^**3**^ **, Shikai Geng** ^**1**^ **, Xiaodong Wang** ^**1**^ **, Liangjing Lu** ^**1**^ **, Shuang Ye** ^**1**^ **, Le Zhang** ^**1,2**^ **, Ting Li** ^**1**^

^1^ Department of Rheumatology, Ren Ji Hospital, Shanghai Jiaotong University School of Medicine, Shanghai, People’s Republic of China ^2^ Department of Pharmacy, Ren Ji Hospital, Shanghai Jiaotong University School of Medicine, Shanghai, China ^3^ Department of Pharmacy, Huangpu Branch, Shanghai Ninth People’s Hospital, Shanghai Jiaotong University School of Medicine, Shanghai, China

The original article has been corrected.

