# Supplementary material for: The effective threshold dose of etanercept in patients with methotrexate-resistant rheumatoid arthritis
Source: Clin Rheumatol. 2023 Jul 7;42(10):2777–86. doi: 10.1007/s10067-023-06659-9 (PMC10497429; doi:10.1007/s10067-023-06659-9)
Supplement: Supplementary file 1 — Supplementary file1 (PDF 368 KB) [file 10067_2023_6659_MOESM1_ESM.pdf]

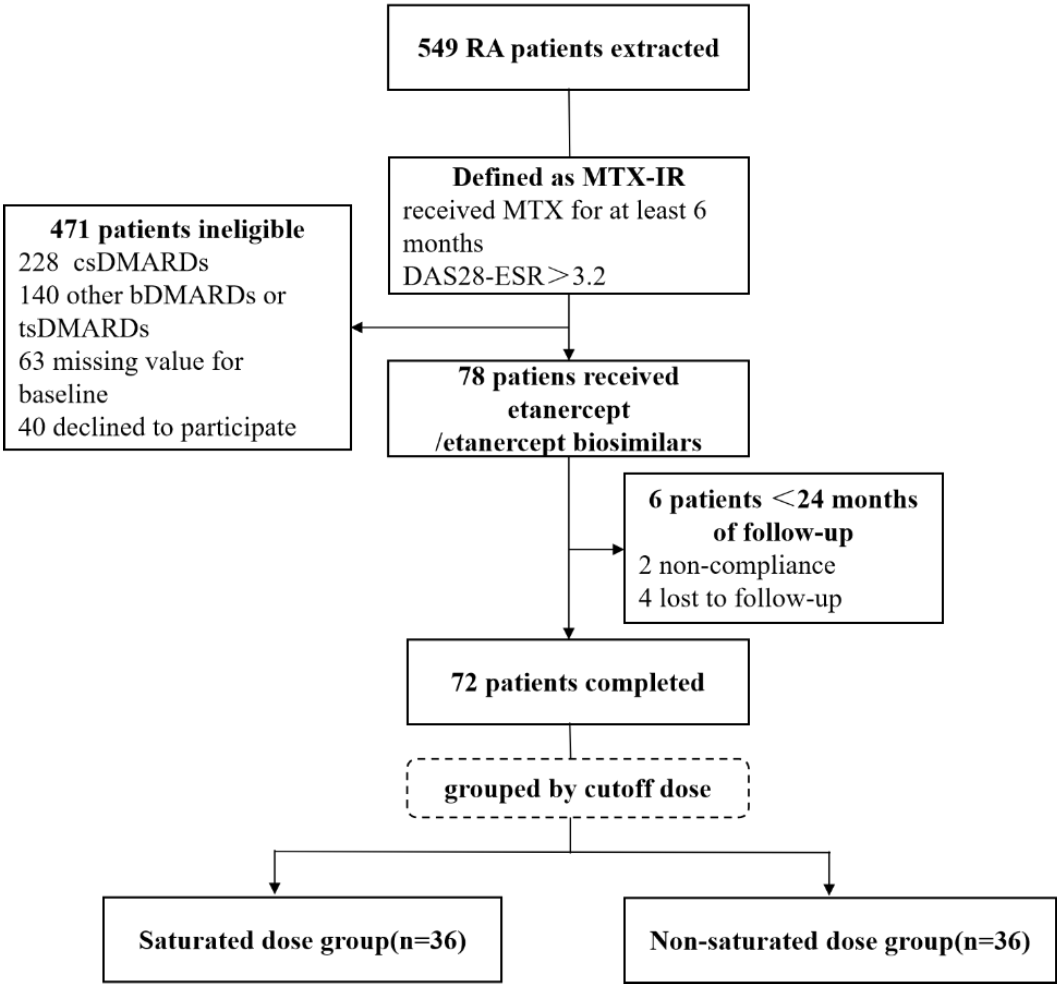

Supplementary Figure S1 Flow chart of our cohort. MTX-IR, methotrexate inadequate response; MTX, methotrexate; RA, rheumatoid arthritis; DAS28-ESR, disease activity score in 28 joints using erythrocyte sedimentation rate; csDMARDs, conventional synthetic disease-modifying antirheumatic drugs; bDMARDs, biological disease-modifying antirheumatic drugs; tsDMARDs, targeted synthetic disease-modifying antirheumatic drugs

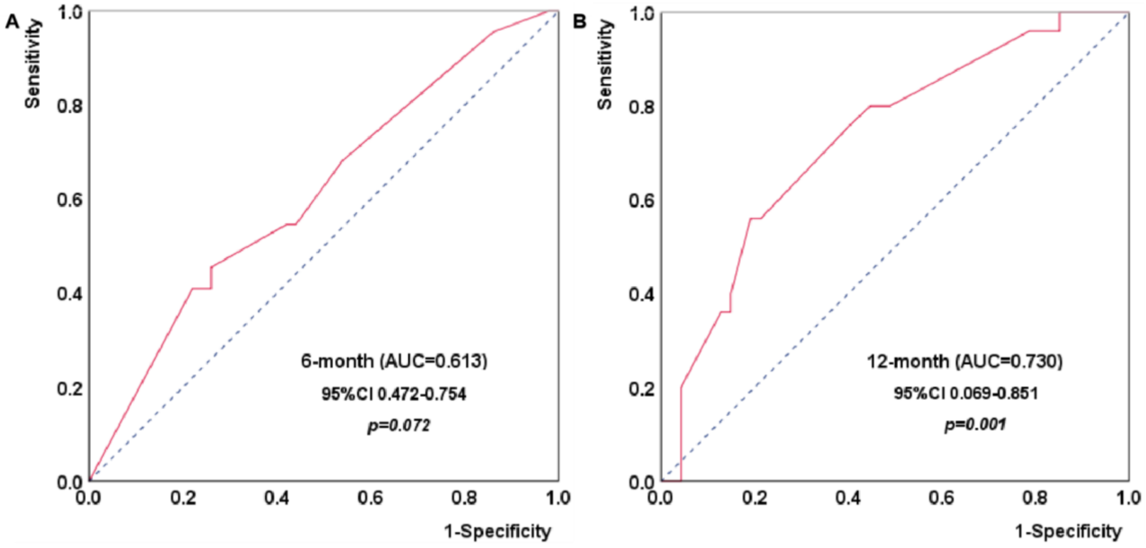

Supplementary Figure S2 ROC curve for remission response at 12 months. ROC, receiver operating characteristics; AUC, area under the curve

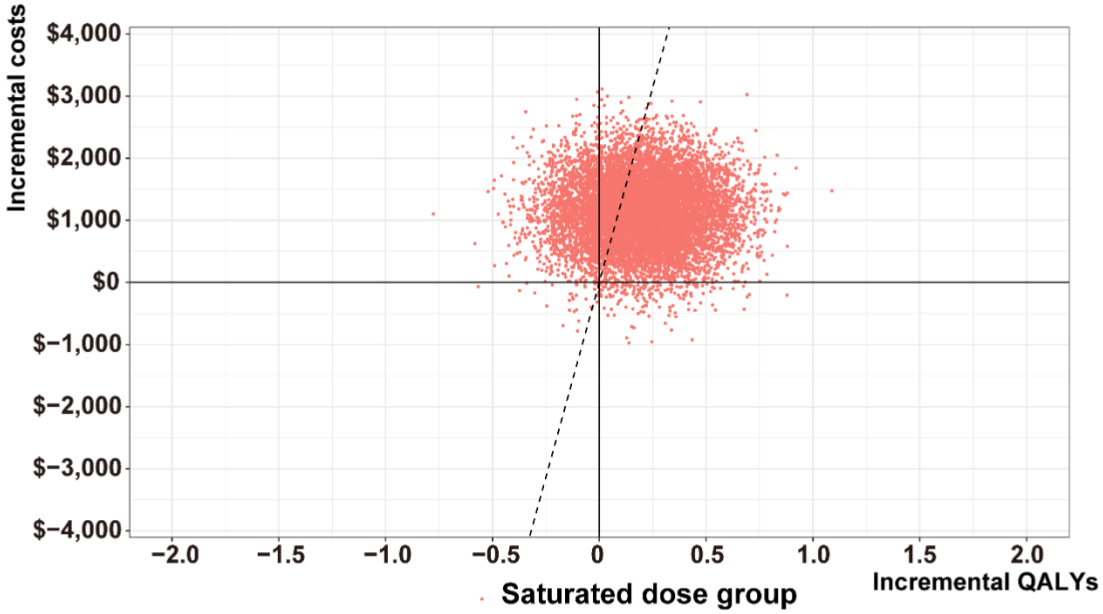

Supplementary Figure S3 Incremental cost-effectiveness plane of two groups. QALY, quality-adjusted life year
