# Supplementary material for: The effective threshold dose of etanercept in patients with methotrexate-resistant rheumatoid arthritis
Source: Clin Rheumatol. 2023 Jul 7;42(10):2777–86. doi: 10.1007/s10067-023-06659-9 (PMC10497429; doi:10.1007/s10067-023-06659-9)
Supplement: Supplementary file 2 — Supplementary file2 (DOCX 35 KB) [file 10067_2023_6659_MOESM2_ESM.docx]

*Supplementary Table S1* Results of the ROC curve to identify the cumulative dose for remission response at 6 and 12 months

| **Time** | **Cumulative dose (mg)** | **Youden index** | **Sensitivity** | **Specificity** |
| --- | --- | --- | --- | --- |
| **6-month** | 699 | 0 | 1 | 0 |
|  | 725 | 0.02 | 1 | 0.02 |
|  | 800 | 0.095 | 0.955 | 0.14 |
|  | 875 | 0.142 | 0.682 | 0.46 |
|  | 950 | 0.105 | 0.545 | 0.56 |
|  | 1025 | 0.125 | 0.545 | 0.58 |
|  | **1075** | **0.195** | **0.455** | **0.74** |
|  | 1125 | 0.149 | 0.409 | 0.74 |
|  | 1175 | 0.189 | 0.409 | 0.78 |
|  | 1201 | 0 | 0 | 1 |
| **12-month** | 899 | 0 | 1 | 0 |
|  | 950 | 0.021 | 1 | 0.021 |
|  | 1025 | 0.043 | 1 | 0.043 |
|  | 1075 | 0.149 | 1 | 0.149 |
|  | 1112.5 | 0.109 | 0.96 | 0.149 |
|  | 1137.5 | 0.13 | 0.96 | 0.17 |
|  | 1175 | 0.173 | 0.96 | 0.213 |
|  | 1250 | 0.311 | 0.8 | 0.511 |
|  | 1325 | 0.332 | 0.8 | 0.532 |
|  | 1400 | 0.353 | 0.8 | 0.553 |
|  | 1475 | 0.356 | 0.76 | 0.596 |
|  | 1550 | 0.347 | 0.56 | 0.787 |
|  | **1625** | **0.369** | **0.56** | **0.809** |
|  | 1675 | 0.251 | 0.4 | 0.851 |
|  | 1725 | 0.211 | 0.36 | 0.851 |
|  | 1775 | 0.232 | 0.36 | 0.872 |
|  | 1850 | 0.157 | 0.2 | 0.957 |
|  | 1950 | 0.077 | 0.12 | 0. 957 |
|  | 2025 | -0.043 | 0 | 0. 957 |
|  | 2075 | -0.021 | 0 | 0.979 |
|  | 2101 | 0 | 0 | 1 |
